# Current noninvasive liver reserve models do not predict histological fibrosis severity in hepatocellular carcinoma

**DOI:** 10.1038/s41598-018-33536-2

**Published:** 2018-10-10

**Authors:** Shu-Yein Ho, Po-Hong Liu, Chia-Yang Hsu, Cheng-Yuan Hsia, Chien-Wei Su, Yi-Jhen He, Yun-Hsuan Lee, Yi-Hsiang Huang, Ming-Chih Hou, Teh-Ia Huo

**Affiliations:** 1 Department of Medicine, Taipei Veterans General Hospital, Taipei, Taiwan; 2 Department of Surgery, Taipei Veterans General Hospital, Taipei, Taiwan; 3 Faculty of Medicine, National Yang-Ming University School of Medicine, Taipei, Taiwan; 4 Institute of Clinical Medicine, National Yang-Ming University School of Medicine, Taipei, Taiwan; 5 Institute of Pharmacology, National Yang-Ming University School of Medicine, Taipei, Taiwan; 6 Department of Internal Medicine, University of Texas Southwestern Medical Center, Dallas, Texas USA; 7 Division of Gastroenterology and Hepatology, University of Michigan, Ann Arbor, MI USA

## Abstract

The Ishak scoring system has been used to stage liver fibrosis. Ten noninvasive liver reserve models were proposed to assess the severity of liver fibrosis, but their performance in hepatocellular carcinoma (HCC) is unknown. We aimed to evaluate the correlation between these models and severity of fibrosis in patients with HCC. A total 464 patients with HCC undergoing surgical resection were retrospectively analyzed. Multivariate logistic regression analysis was performed to determine independent factors associated with advanced fibrosis (Ishak score 4 or higher). There were no significant correlations between all noninvasive models and severity of fibrosis in HCC (p for trend all >0.1). In subgroup analysis, cirrhosis discriminant index (CDS) and Lok’s index in hepatitis B-, and fibrosis index based on 4 factors (FIB-4), CDS and Lok’s index in hepatitis C-associated HCC, best correlated with the severity of liver fibrosis. Low platelet count, prolonged prothrombin time, hepatitis C and multiple tumors were independently associated with advanced fibrosis. Among the 10 models, CDS was the best model to predict cirrhosis. Currently used noninvasive liver reserve models do not well correlate with severity of histological fibrosis in HCC. New noninvasive models are required to improve the predictive accuracy of liver fibrosis in HCC.

## Introduction

Hepatocellular carcinoma (HCC) is a common primary liver cancer worldwide with a rising incidence rate^1,2^. Hepatitis B virus (HBV) infection is the predominant etiology of HCC in Asia and Africa, whereas hepatitis C virus (HCV) infection plays a major role in HCC in Japan and Western countries^3,4^. For early stage HCC, surgical resection and liver transplantation are the cornerstone of curative treatments^3,5,6^. HCC patients are often accompanied with various degrees of liver fibrosis^7^. Severe fibrosis or cirrhosis can lead to serious complications after anti-cancer therapy, and may even cause death from liver failure^8^. In addition, liver fibrosis is also a risk factor for HCC recurrence after curative therapy^9,10^. Therefore, severity of liver fibrosis should be carefully evaluated before definite treatment is given.

Liver biopsy is the standard method to assess liver fibrosis. However, it is an invasive procedure with potentially serious complications that preclude its wide implementation in daily practice. Also, it is not clinically suitable in monitoring the changes of liver fibrosis and cirrhosis over time. Moreover, it could be subject to inter-observer variability and sampling error which can lead to under- or over-staging of disease^11–13^. In comparison with liver biopsy, background fibrosis of liver can be diagnosed more correctly using surgically resected specimens because a large amount of tissue makes sampling variation less likely^14^.

Noninvasive liver reserve models have been introduced to assess the severity of liver fibrosis as a surrogate marker of liver injury. Traditionally, the Child-Turcotte-Pugh (CTP) classification has been widely used to evaluate the severity of liver fibrosis for decades^15^. Other noninvasive models to assess the severity of liver fibrosis include model for end-stage liver disease (MELD) score, aspartate aminotransferase-to platelet ratio (APRI), fibrosis index based on 4 factors (FIB-4), cirrhosis discriminate score (CDS), Göteborg University Cirrhosis Index (GUCI), Lok’s index and King’s score^16–29^. More recently, albumin-bilirubin (ALBI) grade and the platelet-albumin-bilirubin (PABLI) grade were proposed to evaluate the severity of liver fibrosis in HCC^30,31^. However, with all these choices, no study to date has specifically evaluated the relationship and accuracy between noninvasive liver reserve models and the severity of liver fibrosis in HCC. We aimed to investigate the correlation of the currently used noninvasive liver function models and histological fibrosis in HCC patients undergoing surgical resection.

## Material and Methods

### Patients

Patients with newly diagnosed HCC in our hospital during the period from 2009 to 2016 were prospectively identified and retrospectively analyzed. A total 464 patients undergoing surgical resection were enrolled in this study. Their baseline information, including patient’s demographics, etiology of liver disease, performance status, tumoral status, liver functions and serum biochemistry, were comprehensively recorded at the times of diagnosis. The inclusion criteria of surgery were (1) tumor involving no more than three Couinaud segments, (2) CTP class A or B, (3) no main portal vein trunk involvement or distant metastases, and (4) absence of other major diseases that contradict surgical resection^32^. Patients were followed every 3–4 months until death or dropout from follow-up. This study complies with the standard of the Declaration of Helsinki and current ethical guidelines and has been approved by the Institutional Review Broad of Taipei Veterans General Hospital. Waiver of consent was obtained, and patient records/information was anonymized and de-identified before analysis.

### Diagnosis and definition

The pre-operative diagnosis of HCC was based on the findings of typical four-phase multidetector contrast-enhanced dynamic computed tomography (CT) scan or magnetic resonance imaging (MRI)^6,33^, and as all histologically confirmed post-operatively. Patients who were seropositive for hepatitis B antigen (HBsAg), seronegative for anti-HCV antibody, and without a history of alcoholism were classified as HBV-related HCC. HCV-related HCC was defined as seropositive for anti-HCV, seronegative for HBsAg and no history of alcoholism. Dual HBV- and HCV-related HCC was defined as seropositive for HBsAg and anti-HCV^34^. The performance status was assessed by using the Eastern Cooperative Oncology Group Performance scaling ranging from 0 (asymptomatic) to 4 (confined to bed).

### Surgical resection

After the diagnosis was confirmed, patients were reviewed at our multi-disciplinary HCC team for treatment discussion. Share-decisions regarding treatment modalities were made by patients and physicians after individual counseling. Written informed consent was obtained prior to definite treatment. The operative procedures have been previously described in detail^34,35^. The resected liver tissue was sent for gross and microscopic examinations, and the recorded tumor size was based on the largest dimension of the resected specimen.

### Histological analysis

Histology slides of all eligible patients were retrieved and carefully reviewed for tumoral part and non-tumoral part by the pathologists who were blinded to clinical information. The degree of hepatic inflammation and stage of fibrosis in non-tumoral part of the specimen were graded according to the Ishak scoring system. The Ishak staging was defined as the following: 0, no fibrosis; 1, fibrous expansion of some portal area, with or without of short fibrous septa; 2, fibrous expansion of most portal area, with or without short fibrous septa; 3, fibrous expansion of most portal area, with occasional portal to portal bridging; 4, fibrous expansion of portal area with marked bridging as well as portal-central; 5, marked bridging with occasional nodules (incomplete cirrhosis); 6, cirrhosis, probable or definite^36^.

### Grading of 10 noninvasive liver reserve models

The calculation of the 10 noninvasive liver reserve models was based on clinical variables and serum biochemistries at the time of diagnosis. The grading of these models was determined according to published studies^15–18,20–22,24,25,30,31^. Generally, grade 1 indicates no or mild liver fibrosis, and grade 3 shows advanced liver fibrosis or cirrhosis (Table 1).

**Table 1:** Formula and grading of ten noninvasive liver functional reserve models.

Noninvasive blood testing for liver serve makers	Formula		
ALBI, Grade 1/2/3 (<−2.6−2.6-≤−1.39 />−1.39)	(log(Bilirubin[μmol/L]) × 0.66) + (Albumin[g/L] × −0.085)		
APRI, Grade 1/2/3 (<0.5/0.5–1.5/>1.5)	[(AST/upper limit of normal)/Platelet Count (10^9^/l)] × 100		
CTP, A/B/C, grade 1/2/3/ (5–6/7–9/10–15)	Encephalopathy: none = 1, grade 1 or 2 = 2, grade 3 or 4 = 3 Ascites: none = 1, mild to moderate = 2, severe = 3 Bilirubin(mg/dl): <2 = 1, 2–3 = 2, >3 = 3 Albumin(g/dl): >3.5 = 1, 2.8–3.5 = 2, <2.8 = 3 PT sec (INR): <4 (1.7) = 1, 4–6 (1.7–2.3) = 2, >6 (>2.3) = 3		
CDS, Grade 1/2/3 (<4/4–7/>7)	Platelet count (×10^9^/L): >340 = 0; 280–339 = 1; 220–279 = 2; 160–219 = 3; 100–159 = 4; 40–99 = 5; <40 = 6	ALT/AST ratio: >1.7 = 0; 1.2–1.7 = 1; 0.6–1.19 = 2; <0.6 = 3	INR: <1.1 = 0; 1.1–1.4 = 1; >1.4 = 2 CDS is the sum of the above (possible value 0–11)
FIB-4 index, Grade 1/2/3 (<1.45/1.45–3.25/>3.25)	(Age[years] × AST[U/L])/(platelet [10^9^] × ALT[U/L]^1/2^)		
GUCI, Grade 1/2/3 (<0.5/0.5–1.56/>1.56)	[AST/TOPNORMAL AST] × INR × 100/(Platelets × 10^9^)		
Lok’s index, Grade 1/2/3 (<0.5/0.5–0.8/>0.8)	Lok Index = e^(LogOddsLok)^/(1 + e^(LogOddsLok)^) Log Odds Lok = (1.26x AST/ALT) + (5.27 × INR) − (0.0089 × Platelets × 10^9^) − 5.56		
MELD, Grade 1/2/3 (<8/8–12/>12)	10 × ((0.957 × ln(Creatinine)) + (0.378 × ln(Bilirubin)) + (1.12 × ln(INR))) + 6.43		
PABLI, Grade1/2/3(≤−2.53, −2.53 and ≤−2.09, >−2.09)	(2.02 × log_10_ bilirubin) −[0.37 × (log_10_ bilirubin(umol/L))^2^] − 0.04 × albumin (g/L) − 3.48 × log_10_ platelets (10^9^/L) + 1.01 × (log_10_ platelets (10^9^/l))^2^		
King’s score (<7.6/7.6–16.7/16.7)	Age × AST × INR/[platelets (10^9^/l)]		

### Statistical analysis

The *χ*^2^ test or Fisher’s exact test was used to analyze categorical variables, and the Mann-Whitney ranked sum test was used for continuous variables. Factors that showed significant difference in univariate analysis were subjected to multivariate analysis by forward logistic regression to identify independent predictors and determination of odds ratio (OR) and 95% confidence interval (CI). The predictive accuracy of noninvasive models for cirrhosis was determined by calculating the area under receiver operating curve (AUROC)^37^. Spearman’s correlation analysis was used to estimate the correlation between noninvasive liver reserve models and Ishak fibrosis stage in HBV- and HCV-related HCC patients. For all tests, a p < 0.05 was considered statistically significant. All statistical analyses were conducted using the SPSS for Windows version 21 release (SPSS Inc., Chicago, IL, USA).

## Results

### Baseline characteristics

As shown in Table 2, the median age of the study patients was 63 years and 77% were male. The most frequent cause of chronic liver disease was hepatitis B (55%), followed by hepatitis C (16%). Approximately 65% of patients were classified as performance status 0, and 26% had diabetes mellitus. The majority (83%) of patients had single tumor, and 63% of patients had main tumor size larger than 3 cm. In these patients, 118 (26%) and 177 (38%) received lobectomy and bi-segmentectomy, respectively. Another 149 (32%) and 20 (4%) patients received segmentectomy and sub-segmentectomy, respectively. All patients had histologically confirmed HCC and the resected tumors were free of surgical margin. The numbers of patients of each grade in different noninvasive liver reserve models are described in Table 2.

**Table 2:** Baseline characteristics of patients with hepatocellular carcinoma undergoing surgical resection.

Variables	Patients (n = 464)
Age (years, median [interquartile range])	63 [55–71]
Male, n (%)	357 (77)
Etiologies of liver disease	
HBV, n (%)	209 (45)
HCV, n (%)	76 (16)
HBV + HCV, n (%)	21 (5)
Others, n (%)	158 (34)
Diabetes mellitus, n (%)	122 (26)
Performance status (0/1/2–4), n (%)	303/128/33 (65/28/7)
α-fetoprotein (ng/mL) (median, [interquartile range])	18 [4.7–239]
Laboratory values (mean ± SD)	
Alanine aminotransferase (IU/L)	58.5 ± 70.4
Aspartate aminotransferase (IU/L)	56.5 ± 73.5
Albumin (g/dl)	4.0 ± 0.5
Total bilirubin (μmol/dl)	0.88 ± 0.71
Creatinine (mg/dl)	1.18 ± 1.49
Platelets (×10^3^/μL)	171 ± 767
INR of prothrombin time	1.07 ± 0.9
Ishak score (0/1/2/3/4/5/6/), n (%)	18/85/60/55/65/71/110 (4/18/13/12/14/15/24)
Non-invasive liver reserve markers	
ALBI grade (1/2/3), n (%)	274/181/9 (59/39/2)
APRI grade (1/2/3), n (%)	210/184/70 (45/40/15)
CTP classification (A/B-C), n (%)	431/33 (92/8)
CDS grade (1/2/3), n (%)	139/290/35 (30/62/8)
FIB-4 grade (1/2/3), n (%)	104/204/153 (23/44/33)
GUCI grade (1/2/3), n (%)	168/206/90 (36/44/20)
Lok’s index grade (1/2/3), n (%)	269/153/42 (58/33/9)
MELD score (<8/8–12/>12), n (%)	276/152/36 (59/33/8)
PALBI grade (1/2/3), n (%)	275/142/47 (59/31/10)
King’s score (1/2/3), n (%)	82/161/221 (17/35/48)
Tumor nodules (1/2/≥3), n (%)	384/65/15 (83/14/3)
Maximal tumor diameter (<2/2–3/>3 cm), n (%)	79/96/289 (17/20/63)
Extent of hepatic resection	
Sub-segmentectomy, n (%)	20 (4)
Segmentectomy, n (%)	149(32)
Bi-segmentectomy, n (%)	177 (38)
Lobectomy, n (%)	118 (26)

ALBI, Albumin-bilirubin; APRI, Aspartate transaminase-to-Platelet ratio.

CDS, Cirrhosis discriminant index; CTP, Child-Turcotte-Pugh score; FIB-4, Fibrosis-4 score; HBV, hepatitis B virus; HCV, hepatitis C virus; MELD, Model for End-stage liver disease; GUCI, Göteborg University Cirrhosis Index; PALBI, platelet-albumin-bilirubin; SD, standard deviation.

### Correlation of noninvasive liver reserve models and Ishak fibrosis score in HCC

**Figure 1:**
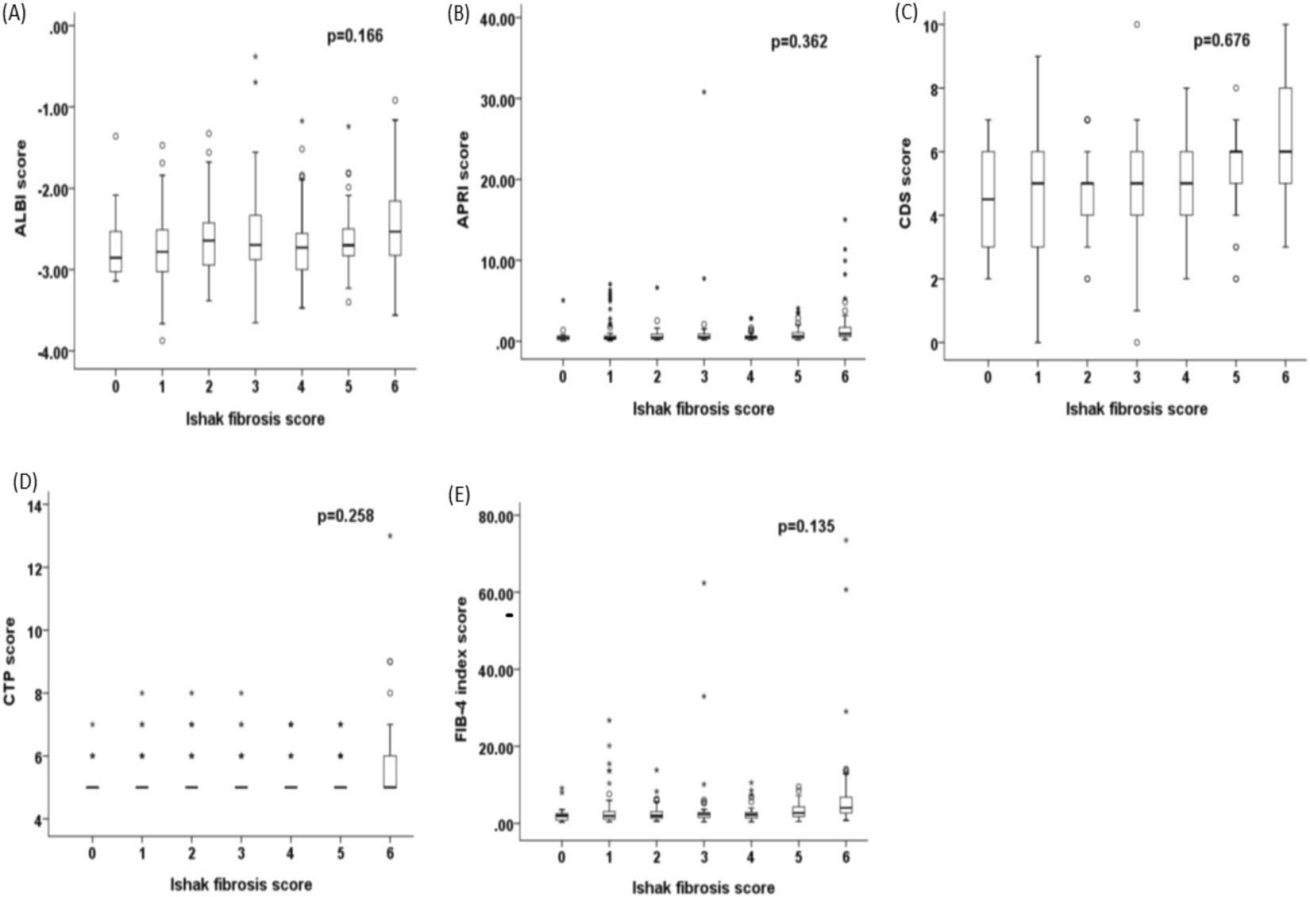
Correlation between (**A**) ALBI, (**B**) APRI, (**C**) CDS, (**D**) CTP, and (**E**) FIB-4 index with Ishak fibrosis score. There is no significant correlation between these models and Ishak score (p values for trend all >0.1). Data were expressed as median (horizontal bars) and 25% to 75% percentile of the distribution (lower and upper margin of the square); the upper and lower vertical bars indicate 90% and 10% percentile of the distribution, respectively. *Indicates extreme values.

**Figure 2:**
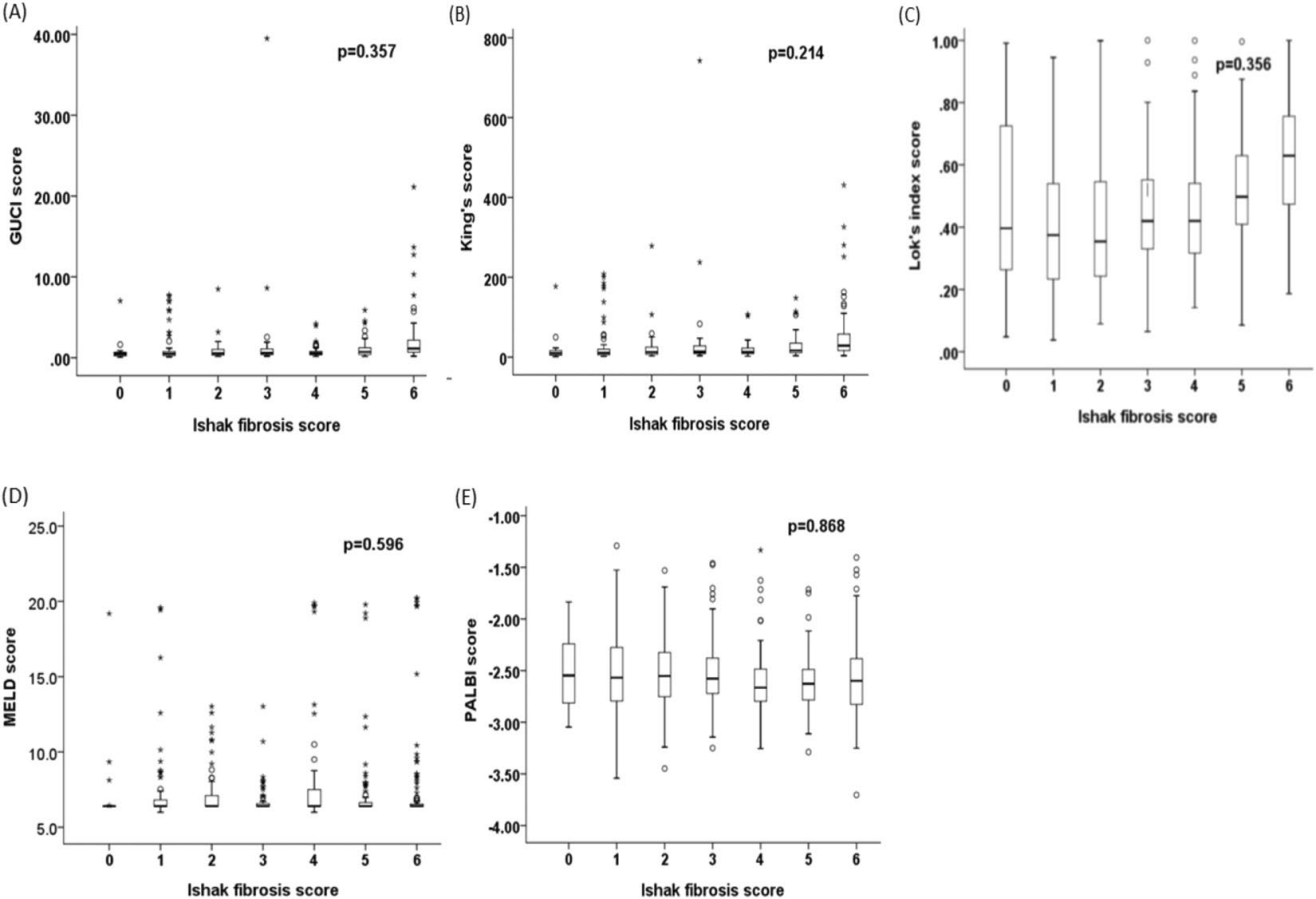
Correlation between (**A**) GUCI, (**B**) King’s score, (**C**) Lok’s index, (**D**) MELD, and (**E**) PALBI with Ishak fibrosis score. There is no significant correlation between these models and Ishak score (p values for trend all >0.2). Data were expressed as median (horizontal bars) and 25% to 75% percentile of the distribution (lower and upper margin of the square); the upper and lower vertical bars indicate 90% and 10% percentile of the distribution, respectively. *Indicates extreme values.

The distributions of Ishak fibrosis scores were as following: score 0, 18 (4%) patients: score 1, 85 (18%) patients; score 2, 60 (13%) patients; score 3, 55 (12%) patients; score 4, 65 (14%) patients; score 5, 71 (15%) patients and score 6, 110 (24%) of patients (Table 2). The relationship of these models and Ishak fibrosis score were assessed. There were no significant correlations between the Ishak score and all 10 (ALBI, APRI, CTP, FIB-4, GUCI, King’s score, MELD, PALBI, CDS and Lok index) models (Figs 1 and 2; p values for trend all >0.1).

### Factors associated with advanced fibrosis in HCC

Two hundred and eighteen (47%) patients with Ishak fibrosis score of 0 to 3 were classified as mild fibrosis group, and 246 (53%) patients with Ishak fibrosis score of 4 to 6 were classified as advanced fibrosis group (Table 3). In comparison with those with mild fibrosis, advanced fibrosis group had a significantly higher prevalence of HCV infection; these patients also had significantly lower platelet count, prolonged international normalized ratio (INR) of prothrombin time and serum bilirubin level >1mg/dl. With regard to tumoral factors, tumor number of 2 or more and tumor size larger than 2 cm were significantly associated with advanced fibrosis. In multivariate logistic regression analysis, anti-HCV-positivity (OR, 1.842; 95% CI, 1.051–3.230; p = 0.033), platelet count less than 150 (x1,000/uL) (OR, 3.397; 95% CI, 2.258–5.111; p < 0.001), INR > 1 (OR, 2.405; 95% CI, 1.444–4.007; p < 0.001), and multiple tumors (OR, 2.018; 95% CI, 1.174–3.469; p < 0.001) were independent risk factors linked with advanced fibrosis (Table 3).

**Table 3:** Risk factors associated with advanced fibrosis in uni- and multivariate analysis.

Variables	Multivariate analysis				
	Mild fibrosis, n = 218	Advanced fibrosis n = 246	*p*	OR (95% CI)	*p*
Age, years mean ± SD	61 ± 13	63 ± 11	0.101		
>60 years	116 (53)	151 (61)	0.091		
Male, n (%)	168 (77)	189(77)	1.000		
HBV, n (%)	93(45)	116 (55)	0.351		
HCV, n (%)	25 (33)	51(67)	0.008	1.842 (1.051–3.230)	0.033
ALT (IU/L) mean ± SD	58 ± 83	59 ± 57	0.948		
>40 IU/L, n (%)	94 (43)	127 (52)	0.077		
AST(IU/L) mean ± SD	57 ± 91	57 ± 53	0.852		
>40 IU/L, n (%)	96(44)	117 (48)	0.456		
Albumin (g/dl) mean ± SD	3.9 ± 0.5	3.9 ± 0.5	0.221		
<3.5 g/dl, n (%)	24 (11)	39 (16)	0.108		
Bilirubin (mg/dl) mean ± SD	0.85 ± 0.76	0.91 ± 0.67	0.421		
>1 mg/dl, n (%)	47(22)	75 (31)	0.034		
Creatinine (mg/dl) mean ± SD	1.05 ± 0.92	1.30 ± 1.84	0.067		
>1 mg/dl, n (%)	66 (30)	74 (30)	1.000		
Platelet(×10^3^/uL) mean ± SD	199 ± 81	146 ± 63	<0.001		
<150, n (%)	56 (26)	142 (58)	<0.001	3.397 (2.258–5.111)	<0.001
INR of prothrombin time mean ± SD	1.05 ± 0.07	1.09 ± 0.09	<0.001	2.405 (1.444–4.007)	0.001
>1, n (%)	154 (71)	215 (87)	0.001		
AFP (ng/ml) mean ± SD	9729 ± 51705	12490 ± 150738	0.784		
>20 ng/ml, n (%)	102 (47)	126 (51)	0.403		
Tumor number mean ± SD	1.16 ± 0.5	1.26 ± 0.5	0.036		
Tumor number ≧2, n (%)	26 (12)	54 (22)	0.005	2.018 (1.174–3.469)	0.011
Tumor size (cm) mean ± SD	5.79 ± 3.8	4.11 ± 2.9	<0.001		
>2cm, n (%)	192 (88)	193 (79)	0.006		

### Noninvasive liver reserve models to predict cirrhosis

The predictive accuracy of the 10 models for cirrhosis (Ishak score of 5 or 6) was assessed by estimating their AUROCs (Table 4). Among these models, CDS had the highest AUROC (0.729), followed by GUCI (AUROC = 0.711) and King’s score (AUROC = 0.709; all p < 0.05).

**Table 4:** Performance of 10 noninvasive liver functional reserve models in predicting cirrhosis (Ishak score 5 or 6).

Noninvasive liver reserve models	AUROC	95% CI	*p*
ALBI	0.594	0.542–0.647	<0.001
APRI	0.706	0.658–0.753	<0.001
CDS	0.729	0.682–0.775	<0.001
CTP	0.561	0.506–0.615	0.028
FIB-4	0.708	0.660–0.756	<0.001
GUCI	0.711	0.664–0.759	<0.001
MELD	0.565	0.513–0.618	0.018
Lok index	0.703	0.655–0.751	<0.001
PALBI	0.467	0.414–0.521	0.233
King’s score	0.709	0.661–0.756	<0.001

AUROC, area under receiver operating curve.

### Correlation of noninvasive models and Ishak fibrosis score according to viral factors

The correlation between liver reserve models and the stage of fibrosis according to viral factors was analyzed (Table 5). There was a relatively high correlation for CDS and Lok index (correlation coefficient, 0.340 and 0.277, respectively, p < 0.001) and the stage of fibrosis specicially in HBV-related HCC. In HCV-related HCC, the correlation was more significant for FIB-4, CDS, Lok index, and APRI with Ishak fibrosis (correlation coefficient: 0.591, 0.546, 0.546, and 0.464, respectively, p < 0.001).

**Table 5:** Correlation of noninvasive liver reserve models and stage of fibrosis in patients with hepatitis B virus (HBV) and hepatitis C virus (HCV) infection.

Noninvasive liver reserve models	HBV (n = 209)		HCV (n = 76)	
	Coefficient	*p*	Coefficient	*p*
ALBI	0.141	0.410	0.323	0.004
APRI	0.066	0.343	0.464	<0.001
CDS	0.340	<0.001	0.546	<0.001
CTP	0.145	0.036	0.233	0.042
FIB-4	0.120	0.083	0.591	<0.001
GUCI	0.076	0.276	0.459	<0.001
King score	0.093	0.179	0.468	0.001
Lok’s index	0.277	<0.001	0.546	<0.001
MELD	0.166	0.016	0.106	0.361
PALBI	−0.113	0.105	0.186	0.109

### Correlation of noninvasive models and tumor burden

We analyzed the correlation between the surrogates of tumor burden (number and size of tumor, serum AFP level) and 10 noninvasive models (Table 6). Single tumor was associated with lower scores of APRI, GUCI, King’s score and PABLI (all p < 0.05), and smaller size of main tumor was linked with lower ALBI, CTP and PABLI score (all p < 0.05). Serum AFP levels tended to be lower with lower scores of APRI, CDS, FIB-4, GUCI, King’s score, Lok index and MELD (all p < 0.05).

**Table 6:** Correlation of tumor burden, serum AFP level and noninvasive liver reserve models.

Noninvasive liver reserve models (mean ± SD)											
	n	ALBI	APRI	CDS	CTP	FIB-4	GUCI	King’s score	Lok index	MELD	PALBI
Number of tumor											
1	385	−2.63 ± 0.5	1.1 ± 2.2	5.19 ± 1.6	5.30 ± 0.7	3.83 ± 6.5	1.31 ± 2.9	32 ± 67	0.48 ± 0.2	8.64 ± 3.4	−2.55 ± 0.4
2	65	−2.68 ± 0.5	1.1 ± 1.6	5.32 ± 1.6	5.43 ± 0.8	3.80 ± 4.0	1.37 ± 2.0	36 ± 54	0.50 ± 0.2	9.03 ± 4.3	−2.63 ± 0.4
≧3	15	−2.58 ± 0.5	1.2 ± 1.1	5.80 ± 1.1	5.47 ± 0.8	4.03 ± 2.5	1.47 ± 1.3	35 ± 30	0.59 ± 0.2	8.66 ± 2.7	−2.51 ± 0.3
p		0.464	0.042	0.208	0.326	0.115	0.032	0.033	0.080	0.639	0.043
Tumor size											
<3 cm	175	−2.67 ± 0.4	1.0 ± 1.3	5.51 ± 1.5	5.28 ± 0.8	3.71 ± 3.9	1.23 ± 1.6	31 ± 42	0.50 ± 0.2	8.69 ± 3.8	−2.63 ± 0.3
3–5 cm	130	−2.68 ± 0.4	1.0 ± 2.0	5.40 ± 1.6	5.23 ± 0.6	3.93 ± 7.2	1.32 ± 2.9	32 ± 64	0.50 ± 0.2	8.31 ± 2.5	−2.60 ± 0.3
>5 cm	160	−2.56 ± 0.5	1.1 ± 2.8	4.78 ± 1.8	5.44 ± 0.8	3.89 ± 7.1	1.32 ± 3.5	34 ± 72	0.47 ± 0.3	9.00 ± 4.0	−2.45 ± 0.4
p		0.049	0.679	<0.001	0.018	0.317	0.727	0.712	0.204	0.267	<0.001
AFP level (ng/ml)											
<20	237	−2.67 ± 0.4	0.9 ± 2.3	3.27 ± 4.8	5.30 ± 0.6	3.27 ± 4.8	1.13 ± 3.1	28 ± 64	0.44 ± 0.2	8.50 ± 3.4	−2.59 ± 0.4
20–200	101	−2.59 ± 0.4	1.4 ± 2.0	5.09 ± 8.3	5.34 ± 0.7	5.10 ± 8.3	1.76 ± 2.7	43 ± 62	0.57 ± 0.2	8.85 ± 3.0	−2.55 ± 0.3
>200	127	−2.61 ± 0.5	1.1 ± 1.6	3.88 ± 6.3	5.35 ± 0.9	3.89 ± 6.3	1.33 ± 2.0	31 ± 46	0.50 ± 0.2	8.90 ± 3.5	−2.51 ± 0.4
p		0.359	<0.001	0.006	0.756	0.006	<0.001	0.003	<0.001	0.021	0.241

## Discussion

Although the CTP and MELD scoring system are widely used in patients with liver diseases, these models are not optimal for evaluating liver fibrosis. Alternatively, noninvasive liver reserve models were proposed to assess the severity of liver fibrosis mainly in patients with chronic hepatitis B or C^18,23,25,28,38–40^. However, the correlation between these models and liver fibrosis in HCC patients is unclear. We have recruited a large cohort of patients undergoing surgical resection to evaluate the degree of liver fibrosis and its association with these models. Surprisingly, our data reveal that none of these models significantly correlate with the severity of histological fibrosis in HCC patients. This finding suggests that the performance of currently used liver reserve models in predicting the severity of liver fibrosis is far from satisfaction. The main possible reason is that the etiologies of fibrosis greatly vary in HCC patients, while these models were generated from patients with distinct clinical characteristics.

We further examined the factors associated with advanced fibrosis, defined as an Ishak score of 4 or higher. Consistent with previous studies^18,20,27,39–41^, low platelet count and prolonged INR, which are known surrogate markers of cirrhosis, are independent predictors of advanced fibrosis. With increasing fibrosis and worsening portal hypertension, there is increased sequestration and destruction of platelets in the enlarged spleen^42^. In addition, progression of liver fibrosis was linked with decreased production of thrombopoietin by hepatocytes, and hence reduced platelet production^43^.

An interesting finding in this study is that the factor of multiple tumors was also independently associated with advanced fibrosis. Previous studies showed that non-cirrhotic HCC patients may have larger tumor size than those with cirrhosis; one of the possible explanations is that non-cirrhotic patients were often diagnosed outside the surveillance program^44,45^. Our earlier study indicated that there was no significant correlation between tumor burden and CTP score and MELD score^46^. However, in the current study, we found that the surrogates of tumor burden tended to associate with the severity of liver fibrosis. Smaller tumor burden may predict a lower score in most noninvasive models except for the comparison between CDS and tumor size. These findings partly explain why these noninvasive models correlate poorly with histological fibrosis, and suggest that the selection of tools in evaluating liver injury for different clinical entities is crucial. Altogether, not only surrogate markers of cirrhosis, the extent of tumoral involvement should also be taken into consideration in predicting non-tumoral part liver damage in HCC.

Noninvasive models, including APRI, CDS, FIB-4, GUCI, King’s score and Lok’s index, were reported to correlate with the degree of fibrosis in HCV-infected patients^20,22–24,27,29,38^. In the current study, we confirm that these 6 models may reflect the severity of liver injury as defined by the Ishak score in HCV-related HCC patients. Additionally, the ALBI score is a new prognostic marker for HCC, and our data indicate that it can also be used to assess liver fibrosis in HCV-related HCC.

Several models, such as APRI, CDS, FIB-4, Lok’s index and King’s score, were also reported to associate with histological fibrosis in HBV-infected patients^23,47,48^. In the current study, however, the correlation is apparent only for CDS and Lok’s index. Our result implies that noninvasive models derived from HCV-infected patients may not be necessarily feasible in predicting histological fibrosis in HBV-related HCC. The pathogenesis of liver fibrosis in chronic hepatitis B is distinct and could be different from that of hepatitis C. Activity of hepatitis B can become quiescent after a period of severe activity, such as recurrent hepatitis flares, or resolution of hepatic necroinflammatory activity following HBV e antigen seroconversion after the development of cirrhosis^49^. In contrast, chronic hepatitis C is a slow but progressive disease with persistent inflammation that ultimately leads to cirrhosis^50^. HCC patients due to HBV or HCV are usually at a late stage of infection and may thus have heterogeneous patterns of liver fibrosis that make prediction with noninvasive models much more difficult.

Noninvasive liver reserve models were also adopted as surrogate markers for discriminating cirrhosis from chronic hepatitis. In accordance with previous studies^20,22,24,38,39,48^, we found that APRI, CDS, FIB-4, GUCI, Lok’s index and King’s score had moderate power to predict cirrhosis in HCC, with an AUROC between 0.703–0.729. Among these models, CDS was identified as the best model to predict cirrhosis. Given so, the predictive accuracy is considered not satisfactory, and new models are needed to refine the predictive ability for cirrhosis in HCC.

This study has a few limitations. Firstly, in this single-hospital study, the major etiology of HCC is HBV infection. This feature is apparently different from Western counties where HCV infection is the predominant etiology of HCC. Secondly, our hospital is a tertiary medical center. Therefore referral bias cannot be completed avoided. Lastly, since this study is retrospective in nature, external validation from independent patient cohorts is required.

In conclusion, the currently used noninvasive liver reserve models do not well correlate with the severity of histological fibrosis in HCC. Among these models, CDS is more accurate in predicting the presence of cirrhosis. Different models should be used for HCC patients with different viral etiology. In addition to traditional cirrhosis surrogates, the extent of tumoral involvement and viral factor are crucial determinants that contribute to liver injury. We advocate that new models should be explored to enhance the predictive ability for liver fibrosis in the setting of HCC.
